# Disease risk prediction with rare and common variants

**DOI:** 10.1186/1753-6561-5-S9-S61

**Published:** 2011-11-29

**Authors:** Chengqing Wu, Kyle M Walsh, Andrew T DeWan, Josephine Hoh, Zuoheng Wang

**Affiliations:** 1 Department of Epidemiology and Public Health, Yale University, 60 College Street, New Haven, CT 06510, USA

## Abstract

A number of studies have been conducted to investigate the predictive value of common genetic variants for complex diseases. To date, these studies have generally shown that common variants have no appreciable added predictive value over classical risk factors. New sequencing technology has enhanced the ability to identify rare variants that may have larger functional effects than common variants. One would expect rare variants to improve the discrimination power for disease risk by permitting more detailed quantification of genetic risk. Using the Genetic Analysis Workshop 17 simulated data sets for unrelated individuals, we evaluate the predictive value of rare variants by comparing prediction models built using the support vector machine algorithm with or without rare variants. Empirical results suggest that rare variants have appreciable effects on disease risk prediction.

## Background

The potential of common genetic variants detected from genome-wide association studies to predict the risk of complex diseases has been investigated in a steadily increasing number of empirical studies. So far, these studies generally show limited predictive value of genetic factors [1-4]. This finding might be due to the use of only a limited number of confirmed susceptibility loci. However, a prediction model built using the support vector machine (SVM) algorithm achieves improved performance when a large number of single-nucleotide polymorphisms (SNPs) are included in the prediction models [5].

Recently, substantial advances have taken place in developing new sequencing technologies (e.g., ABI/SOLiD, Roche/454, and Illumina/Solexa) [6]. Sequencing of candidate genes, exons, or whole genomes will allow the identification of rare susceptibility variants that may have stronger effects on disease susceptibility [3]. Although more and more rare variants have been found to be associated with complex diseases [7,8], it is still unclear whether rare variants will improve disease risk prediction.

The aim of this study is to determine whether rare variants provide valuable predictive information beyond that provided by common variants and environmental covariates alone. To this end, we examine the effect of adding collapsed rare SNPs to prediction models that include both environmental covariates and common genetic variants in the Genetic Analysis Workshop 17 (GAW17) simulation data sets. The prediction models are built using an SVM algorithm [5,9,10], which uses biomarkers that have reached a predefined statistical threshold for association with the disease. As discussed by Wei et al. [5], the SVM-based risk prediction algorithm, which is inherently capable of handling intermarker correlation structure, can generate consistent and robust prediction models across different data sets.

## Methods

### Data

The first data set of GAW17 consists of a collection of 697 unrelated individuals from the 1000 Genomes Project. There are 200 replicates of simulated trait information and a number of nongenetic covariates such as age, sex, and smoking status. SNP genotypes were obtained from the sequence alignment files provided by the 1000 Genomes Project for their pilot3 study [11]. Included are 24,487 autosomal SNPs from 3,205 genes.

### Risk prediction models

To assess the effect of rare variants on global disease risk prediction, we consider prediction models built using an SVM algorithm. The SVM is one of the popular classifiers in the field of machine learning and delivers state-of-the-art performance in a wide variety of biological applications [5]. In essence, the SVM is a supervised learning method that produces nonlinear boundaries by constructing a linear boundary in a transformed version (kernel function) of the feature space (SNP genotypes); thus it achieves maximum separation between two classes of subjects (case group vs. control group). Unlike traditional regression-based methods, the SVM is particularly useful in classifying high-dimensional data by allowing more input features, such as SNPs or genes. We include in the prediction model those genetic variants with *p*-values less than a prespecified threshold from association analysis, with adjustment for covariates. Here, rare variants are defined as SNPs with minor allele frequency (MAF) less than 5% [12,13].

The association between disease and common SNPs (MAF ≥ 5%) is evaluated using Fisher’s exact test by comparing allele counts between case subjects and control subjects. SNPs with *p*-values less than a prespecified threshold (e.g., 1.0 × 10^−3^) are used for disease risk assessment in the next step. For the analysis of rare variants (MAF < 5%), SNPs are first collapsed by the presence or absence of minor alleles within each gene in each individual [14-17]. For each gene, we consider two sets of rare SNPs: the set of all rare variants and the set of all nonsynonymous rare variants. The collapsing approach is applied to each of the two sets. For each set of variants, the disease status is modeled in a logistic regression framework as a function of the presence or absence of a rare allele in the SNP set. Genes reaching a predefined statistical threshold are included in the risk prediction model. For a gene for which both rare variant sets reach the threshold, the set with the smaller Akaike information criterion (AIC) is selected to model the effect of rare variants in the gene. The *p*-value threshold used to select variants ranged from 1.0 × 10^−5^ to 0.01.

The SVM training algorithm is applied to these variants and to the covariates Age, Sex, and Smoking status. The genotype data for common SNPs are coded 0, 1, or 2, reflecting the number of minor alleles. Rare variants are coded 1 or 0, corresponding to the presence or absence, respectively, of minor alleles within each gene. Prediction models are built to discriminate between case subjects and control subjects. The risk prediction model is built using the SVM algorithm in the training data set, and the prediction error of the model is assessed in the validation data sets.

To evaluate the predictive value of rare variants, we conducted two experimental studies. In the first experiment, the set of SNPs included in the risk prediction model was selected from the first trait replicate, and the prediction model was built on the same data set. Prediction error was assessed on the remaining 199 trait replicates. In the second experiment, for each trait replicate we randomly divided the data into a training set and a validation set. SNP selection and risk prediction models were performed on the training set, and prediction error was estimated from the validation set. We repeated this procedure in each of the 200 trait data sets. In this second experiment, the size of the training set took values from 300 to 600, with an increment of 100.

We used the R package e1071 to build the risk prediction models. This package is an interface to the LIBSVM implementation of the SVM algorithm (current version 3.0, http://www.csie.ntu.edu.tw/~cjlin/libsvm). We trained the soft-margin linear SVM classifiers [18] in the training data sets using the SVM penalty parameter *C* = 1, the default value of the R package.

To evaluate the performance of risk prediction models, we applied receiver operating characteristic (ROC) curve analysis to the validation data sets. The ROC is a widely used tool to evaluate the discrimination ability of a binary classifier. In ROC analysis, the discriminatory power of the prediction model is usually measured as the area under the ROC curve (AUC value). This is the probability that a randomly chosen positive sample will have higher predicted risk than that of a randomly chosen negative sample. We compared the AUC values of prediction models combining both common and rare variants with the AUC values of models incorporating only common variants.

## Results

Seven *p*-value cut points were selected from 1.0 × 10^−5^ to 0.01. All SNPs reaching the predefined threshold were included in the prediction models. We evaluated the prediction potential of rare variants by comparing models including rare variants with models without rare variants.

Table 1 shows the AUC values of models with both common and rare variants and of models with common variants, the differences in AUC values between models, and 95% confidence intervals of these AUC value differences for the first experiment. The AUC values and the AUC value differences are similar across different *p*-value thresholds, despite the fact that a higher threshold allows more genetic factors in the prediction model. This suggests that adding less significant SNPs does not provide sufficient improvement in discrimination power. In comparing the AUC values between models with and without rare variants, we found that incorporation of rare variants slightly improved the prediction. The improvement was statistically significant if we compared the two AUC values using a paired *T*-test. However, the magnitude of the improvement was relatively small (<0.4%).

**Table 1:** AUC values for models with and without rare variants in the first experiment

Variant selection *p*-value threshold	Common variants only	Common and rare variants	AUC value difference with 95% confidence interval
0.01	0.8057	0.8088	0.0031 (0.0028, 0.0034)
0.005	0.8063	0.8088	0.0025 (0.0023, 0.0027)
0.001	0.8056	0.8076	0.0020 (0.0019, 0.0021)
5.0 × 10^−4^	0.8062	0.8080	0.0018 (0.0017, 0.0019)
1.0 × 10^−4^	0.8075	0.8093	0.0017 (0.0017, 0.0018)
5.0 × 10^−5^	0.8075	0.8092	0.0017 (0.0017, 0.0018)
1.0 × 10^−5^	0.8079	0.8096	0.0017 (0.0016, 0.0017)

Prediction models were built on the first phenotype replication.

In the first experiment, the prediction model was built on the first trait replicate. We also evaluated the performance of prediction models using other trait replicates as a training set. Models built on different replicates could choose quite different sets of genetic variants, but the results of these models were similar to the results shown in Table 1.

Table 2 presents the comparison results for the second experiment. Within a fixed size of the training set, the AUC value is larger for most of the prediction models as the *p*-value threshold increases. Therefore prediction models perform better with more variants included in the model. However, this relationship is not apparent when the size of the training set is 300. With such a small training set, the estimation of association between disease and variants is not accurate. A larger *p*-value threshold may result in a higher proportion of false-positive variants included in the prediction model, thus leading to smaller AUC values.

**Table 2:** AUC values for models with and without rare variants in the second experiment

Variant selection *p*-value threshold	Common variants only	Common and rare variants	AUC value difference with 95% confidence interval
Size of training set = 300			
0.01	0.7901	0.8442	0.0541 (0.0520, 0.0562)
0.005	0.8014	0.8364	0.0350 (0.0329, 0.0372)
0.001	0.8199	0.8437	0.0239 (0.0227, 0.0250)
5.0 × 10^−4^	0.8185	0.8429	0.0244 (0.0234, 0.0255)
1.0 × 10^−4^	0.8191	0.8333	0.0142 (0.0136, 0.0148)
5.0 × 10^−5^	0.8177	0.8310	0.0133 (0.0128, 0.0139)
1.0 × 10^−5^	0.8124	0.8279	0.0154 (0.0150, 0.0159)
Size of training set = 400			
0.01	0.8315	0.8857	0.0542 (0.0524, 0.0561)
0.005	0.8348	0.8724	0.0376 (0.0362, 0.0391)
0.001	0.8346	0.8590	0.0244 (0.0236, 0.0253)
5.0 × 10^-4^	0.8301	0.8564	0.0263 (0.0256, 0.0270)
1.0 × 10^-4^	0.8247	0.8393	0.0146 (0.0143, 0.0150)
5.0 × 10^-5^	0.8219	0.8355	0.0135 (0.0132, 0.0139)
1.0 × 10^-5^	0.8157	0.8313	0.0156 (0.0152, 0.0159)
Size of training set = 500			
0.01	0.8616	0.9182	0.0565 (0.0551, 0.0579)
0.005	0.8572	0.8966	0.0394 (0.0384, 0.0404)
0.001	0.8443	0.8686	0.0244 (0.0238, 0.0249)
5.0 × 10^−4^	0.8373	0.8636	0.0263 (0.0258, 0.0268)
1.0 × 10^−4^	0.8280	0.8422	0.0142 (0.0139, 0.0145)
5.0 × 10^−5^	0.8245	0.8378	0.0133 (0.0130, 0.0135)
1.0 × 10^−5^	0.8177	0.8331	0.0154 (0.0152, 0.0157)
Size of training set = 600			
0.01	0.8850	0.9470	0.0619 (0.0610, 0.0629)
0.005	0.8730	0.9149	0.0418 (0.0411, 0.0425)
0.001	0.8510	0.8754	0.0244 (0.0240, 0.0248)
5.0 × 10^−4^	0.8420	0.8682	0.0262 (0.0259, 0.0265)
1.0 × 10^−4^	0.8303	0.8443	0.0140 (0.0138, 0.0141)
5.0 × 10^−5^	0.8259	0.8393	0.0133 (0.0132, 0.0135)
1.0 × 10^−5^	0.8188	0.8342	0.0155 (0.0154, 0.0156)

Prediction models were built on a randomly selected training data set for each replicate.

In the second experiment, the differences between the AUC values of models with rare variants and models without rare variants were significant at the 0.05 level, and the differences were much larger than the differences in the first experiment. The largest AUC difference between two models was more than 6%, suggesting a great potential for the improvement of prediction models through incorporation of rare variants.

## Discussion and conclusions

By using prediction models built on the GAW17 simulated data sets and using the SVM algorithm, we conducted two experiments to assess the value of rare variants in complex disease risk prediction. In our studies, including rare variants marginally improved the classification of risk prediction models in the first experiment and substantially improved the classification in the second experiment. In both experiments, rare variants had an appreciable effect on disease risk prediction.

In the SVM literature, two kernel functions are commonly used: the linear kernel:

*k*(**x**, **y**) = **x′y** (1)

and the radial kernel:(1)

We applied both the radial kernel and the linear kernel in our two experiments. The predictive values contributed by rare variants were similar between the two kernels. The results presented in this paper are limited to the linear kernel for its good interpretability [5].

In addition, we used different penalty parameters, ranging from 0.001 to 1000, to build the prediction models. Although the SVM algorithm tends to assign different weights for risk factors under different penalty parameters, the performance of the prediction models are similar for different penalty parameters. Results for the penalty parameter *C* = 1 are shown in this paper.

The AUC value is one of the popularly used statistics for model comparison. We also computed other measurements of discriminatory power for prediction models, such as accuracy, true-positive rate, false-positive rate, positive predictive value, and negative predictive value. The difference between the two types of prediction models have similar patterns to the results for the AUC value (data not shown).

## Competing interests

The authors declare that there is no competing interest**.**

## Authors’ contributions

CW and ZW designed the study, and CW performed the statistical analysis and drafted the manuscript. ZW participated in the design of the study and provided critical revisions of the manuscript. KMW and ATD participated in designing the study, reviewing and editing the manuscript. JH directed the study and revised the manuscript. All authors read and approved the final manuscript.
